# Selected Physical and Spectroscopic Properties of TPS Moldings Enriched with Durum Wheat Bran

**DOI:** 10.3390/ma15145061

**Published:** 2022-07-20

**Authors:** Maciej Combrzyński, Agnieszka Wójtowicz, Anna Oniszczuk, Dariusz Karcz, Jarosław Szponar, Arkadiusz P. Matwijczuk

**Affiliations:** 1 Department of Thermal Technology and Food Process Engineering, University of Life Sciences in Lublin, 20-612 Lublin, Poland; agnieszka.wojtowicz@up.lublin.pl; 2 Department of Inorganic Chemistry, Medical University in Lublin, 20-093 Lublin, Poland; anoniszczuk@o2.pl; 3 Department of Chemical Technology and Environmental Analytics (C1), Faculty of Chemical Engineering and Technology, Cracow University of Technology, 31-155 Kraków, Poland; dariusz.karcz@pk.edu.pl; 4 Toxicology Clinic, Clinical Department of Toxicology and Cardiology, Stefan Wyszyński Regional Specialist Hospital, Medical University of Lublin, 20-718 Lublin, Poland; szponar.jarek@gmail.com; 5 Department of Biophysics, University of Life Sciences in Lublin, 20-950 Lublin, Poland

**Keywords:** extrusion cooking, durum wheat bran, thermoplastic starch moldings, mechanical and structural properties, color profile, ATR/FTIR spectroscopy

## Abstract

The impact of the amount of durum wheat bran additive used on the selected structural, mechanical, and spectroscopic properties of thermoplastic starch moldings was examined in this study. Bran was added to corn starch from 10 to 60% by weight in the blends. Four temperature settings were used for the high-pressure injection: 120, 140, 160, and 180 °C. The highest value of elongation at break (8.53%) was observed for moldings containing 60% bran. Moreover, for these moldings, the tensile strength and flexural strength were lower (appropriately 3.43 MPa and 27.14 MPa). The highest deformation at break (1.56%) were obtained for samples with 60% bran and injection molded at 180 °C. We saw that higher bran content (50 and 60%) and a higher injection molding temperature (160 °C and 180 °C) significantly changed the color of the samples. The most significant changes in the FTIR spectra were observed at 3292 and 1644 cm^−1^ and in the region of 1460–1240 cm^−1^. Moreover, notable changes were observed in the intensity ratio of bands at 1015 and 955 cm^−1^. The changes observed correspond well with the amount of additive used and with the injection temperature applied; thus it may be considered as a marker of interactions affecting plasticization of the material obtained.

## 1. Introduction

Over the last several decades, plastic has become truly ubiquitous in virtually all areas of human existence. Removing plastic in the production of packaging, toys, cars, medicinal products, etc., has become almost unthinkable. However, although the use of such materials is undeniably highly convenient, significant negative aspects thereof are also observed. Indeed, the problem of processing used plastic packaging is one of the central issues faced by 21st century societies. The world’s population has grown considerably in recent years, which, along with globalization has led to increased consumption, which has enhanced the amounts of packaging materials (generally plastics) needed. It is generally accepted that the end of the 20th century brought about significant changes in the development of the packaging industry, which started to pursue additional, entirely new goals [1]. This change was triggered by the popularization of hypermarkets, improvement of sales systems relying on bar code labelling, and a growing demand for disposable packaging. Increasingly, apart from the price, quality, and attractive design, the perceived eco-friendliness of the product, including its packaging, has become a selling point.

When designing new packaging form factors, it is important to anticipate future sentiments of customers. Faced with rows of fully stacked shelves, the modern shopper tends to choose products whose image evokes specific emotions [2]. The widely prevalent fears for the condition of the natural environment have inspired a growing interest in materials capable of biodegradation after a brief period of use. The current pro-environmental sentiment, facilitated by growing eco-awareness of many societies, has effectively encouraged studies on alternative, biodegradable materials. The future development of the packaging industry is likely to focus on two key concepts: biodegradation and natural materials. Global pro-environmental efforts, statutory support, and growing consumer interest, coupled with the decreasing costs of petrochemical alternatives, are all factors facilitating the dynamic development in the biocomposite and bioplastic market [3,4].

Legal regulations now favor solutions that are environmentally friendly and fabricated from renewable raw materials, particularly in the packaging industry. As such, biodegradable, starch-based biomaterials, are becoming a viable alternative for traditional composites, despite their higher production costs. In recent years, irrespective of the economic crisis, the market for biodegradable packaging has continued to grow very dynamically [5,6,7].

Considerable attention is devoted to biodegradable polymers that relatively quickly decompose after use and consequently pose no environmental pollution [8]. Various natural, starch-based materials form an important group among such rapidly degradable biomaterials [9,10,11]. In order to be suitable for use as biodegradable packaging materials, unprocessed starch must be transformed into thermoplastic starch (TPS). A biopolymer is obtained once starch is mixed with a plasticizer (often glycerol) to prevent the material’s liquification at temperatures below the decomposition temperature of the starch, e.g., in the process of extrusion. The properties of TPS depend on the botanical origin of starch, or more specifically, on the relative ratio of its two main components: linear amylose and branched amylopectin. There have been numerous studies exploring the impact of amylose and amylopectin content on the properties of starch-based materials [12,13,14].

It has been observed that the addition of wheat bran, a waste product generated during the process of grinding various grains, can affect the mechanical properties of the final bioproduct, e.g., foil: the value of the elastic storage modulus E is increased (energy stored in the material, representing the product’s elastic properties), DSC shows higher gelation temperatures and lower melting temperatures compared with pure foil without fillers. As revealed by IR data, interactions between the starch and the filler increase the availability of the hydroxyl groups involved in the dynamic exchange with water [15]. Apart from foil, rigid forms can also be used in the production of, e.g., flowerpots, disposable plates, or starch foam fillers for transport packaging. The use of wheat bran also significantly influences the texture and structure of starch-based materials, which connects to modifications in terms of mechanical properties [16]. ATR/FTIR techniques have been successfully applied to biopolymers, for example, to study changes in the surface topography of starch films enriched with selected functional additives [17].

The present study is aimed at an analysis of the physical and spectroscopic properties of new types of biodegradable starch-based moldings with various level of durum wheat bran enrichment. For this purpose, the selected structural, mechanical and color properties of TPS moldings were tested. Additionally, Attenuated Total Reflectance/Fourier Transform Infrared Spectroscopy (ATR/FTIR) measurements allowed us to study changes taking place in the selected biopolymer samples at the molecular level.

## 2. Materials and Methods

### 2.1. Samples Preparation

MERIZET corn starch 100 (moisture 14.0%, amylose 28.2%, and amylopectin 71.8%, residual proteins 0.26%) produced by Segezha (Ashbourne, Ireland), mixed with 20% of 99% purity glycerol (Odczynniki Chemiczne, Lublin, Poland) was used as the basic raw material. Grain milling waste, durum wheat bran (Duragold durum wheat variety, degree of granulation over 560 µm; moisture 12.5%; protein 16.2%; fiber 38.5%; fat 5.6%; and total carbohydrates 52.3%) was purchased from PZZ Lubella GMW Sp. z o.o. Sp.k. (Lublin, Poland). The amount of durum wheat bran in the prepared blends was set to 10, 20, 30, 40, 50, and 60% of the sample mass (mixture of corn starch and glycerol); samples without additives (only corn starch and glycerol) were prepared for comparison as control. Corn starch and the additives were mixed in a laboratory ribbon mixer for 20 min (without additional water). Next, the blends were stored for 24 h in plastic bags before testing. The prepared samples were mixed again for 10 min, prior to the extrusion cooking process, which guaranteed a loose structure of the compounds.

### 2.2. The Extrusion Cooking of TPS Granulates

The sample preparation process was conducted in two stages. In the first stage, extrusion cooking of the prepared raw materials blends was performed to obtain thermoplastic starch granulate. A modified TS-45 single screw extruder cooker (ZMCh Metalchem, Gliwice, Poland) with L/D = 18/1 was used during the extrusion cooking process. The machine was equipped with an additional barrel cooling section (L/D = 6/1, cooling agent mixture of glycerol 50% and water 50%, cooled by external chiller) and a forming die with a 3 mm hole diameter. The granulate was produced using the extruders’ screw set to 80 rpm. The extrusion cooking temperature was set in the range of 85–120 °C and maintained appropriately by adjusting the intensity of the flow of cooling liquid. The processing temperature was measured with thermocouples installed along the barrel; the results were recorded.

### 2.3. The Injection Molding Process

**Figure 1:**
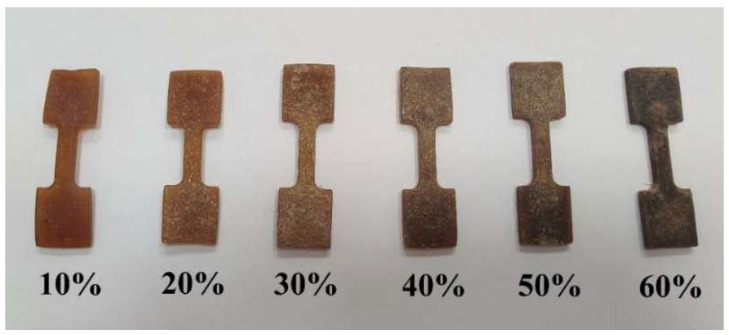
Starch-based moldings with various ratios of durum wheat bran addition.

In the second stage of the experiment, the prepared thermoplastic starch granulates enriched with durum wheat bran were used to produce standard samples in the form of biocomposite moldings suitable for the testing of mechanical properties (Figure 1). The moldings were prepared by employing a high-pressure injection molding process, using a ARBURG 220H90-350 injection molding machine set to L/D = 20.5 (Lossburg, Germany). The material was initially injected at 120 °C, and the temperature was subsequently increased in 20 °C increments to reach 180 °C.

### 2.4. Moisture Content Stability

All the samples (blends before extrusion cooking; granulates before injection process, and final specimens before being tested) were stored in closed plastic bags under laboratory conditions, e.g., room temperature 22 °C, air humidity 65%.

Before testing of TPS moldings, the moisture content of each sample group (bran addition/injection molding process temperature) was standardized by applying the standard air-drying method at 130 °C and 1 h drying. Moldings with moisture content that differed by more than 1% in each sample group were rejected and not tested.

During the study, it was observed that both the temperature of TPS injection and the percentage ratio of durum wheat bran enrichment significantly influenced the pressure of biopolymer injection. The choice of the appropriate biomaterial injection pressure is of paramount importance as it directly impacts the quality, as well as the physical and practical properties of the end product. Where the biopolymer injection temperature was too low, the mold was not properly filled, causing multiple defects and disqualifying the product. In contrast, excessive injection pressure caused the emergence of undesirable risers, as well as problems with extracting the moldings from injection molds.

### 2.5. Structural Analysis of TPS Moldings

Biopolymers samples were cut into 2 × 4 × 1 mm pieces using cutting device Dremel 4000 (Bosch Power Tools B.V., Robert Bosch Power Tools GmbH, Leinfelden-Echterdingen, Germany) from the center part of the specimen. Structure was analyzed by ZEISS Stemi 508 Greenough Stereo Microscope with 8:1 Zoom (Carl Zeiss Sp. Z o.o., Poznań, Poland) with magnification of ×2.5 and external source of light.

### 2.6. Mechanical Properties of TPS Granulates and Moldings

To determine the selected mechanical properties of the samples, the moldings underwent tensile and flexural strength testing. The examination of the mechanical properties of the biopolymer moldings (tensile strength) was performed using a Zwick/Roell BDO-FBO 0.5^TH^ universal testing machine (Ulm, Germany) fitted with suitable extension equipment in the form of stretching jaws and a 0.5 kN head in accordance with the methodology described by Anuar et al. [18] with own modification. The tensile test speed was 5 mm·min^−1^. To determine the cross-sectional area of the sample, the thickness and width of the moldings were measured in three points of the samples’ working area, with an accuracy of 0.01 mm, using a micrometer. The TestXpert ver. 10.11 software (Zwick Roell Polska Sp.zoo. Sp.k., Wrocław, Poland)was employed to facilitate registration and processing of the results of the tensile strength tests. The measurements were performed in 10 replications for each sample type.

The examination of the mechanical properties of moldings (flexural strength) was also performed using a Zwick/Roell BDO-FBO 0.5^T^H universal testing machine (Ulm, Germany) fitted with suitable three-point bending equipment and a 0.5 kN head in accordance with the methodology provided in PN-EN ISO 178:2019-06452 with own modification [19]. The test speed was 5 mm·min^−1^. The strength tests entailed the pointwise application of load onto a biopolymer molding supported at the ends, perpendicularly to its lengthwise axis. The load was applied at a constant rate to measure the samples’ flexural strength (threshold stress) and deformation at break. To determine the cross-sectional area of the sample, the thickness and width of the moldings were measured at three points of the samples’ working area, with an accuracy of 0.01 mm, using a micrometric screw. The examinations were processed using the TestXpert ver. 10.11 software (Zwick Roell Polska Sp.zoo. Sp.k., Wrocław, Poland) to facilitate the registration and processing of results. The measurements were performed in 10 replications for each sample type.

### 2.7. Measurement of Moldings Color

The color of starch-based moldings was determined using a NR20XE colorimeter (District, Shenzhen, China), based on the CIE-Lab system. The L*, a*, and b* coordinates were evaluated in five replications for each sample. The L* parameter lightness ranged from 0 (black) to 100 (white). The coordinate a* described changes from red if positive to green if negative, whereas b* described the range between yellow if positive and blue if negative. Additionally, the total color change index ΔE was calculated, based on Bouasla, Wójtowicz and Zidoune [20].

### 2.8. Infrared Spectra Measurements

For the studied samples, spectra were measured using an IRSpirit spectrometer manufactured by Shimadzu (Tokyo, Japan). An Attenuated Total Reflection (ATR) apparatus was employed in the form of a ZnSe crystal with adequate geometry (i.e., truncated at 45°) to allow for 20-fold internal reflection of the absorbed beam. Before and after each measurement, the crystal was carefully cleaned using ultra-clean and clear solvents purchased from Sigma-Aldrich (Darmstadt, Germany). For 2 h before the measurement, as well as during the same, a neutral gas (N_2_) atmosphere was maintained inside the measurement chamber. Spectra were registered in the range from 450 to 3750 cm^−1^ at a good resolution of 0.5 cm^−1^. Subsequently, the spectral data were analyzed and processed through Grams/AI software by ThermoGalactic Industries (Waltham, MA, USA). All spectra were measured at room temperature.

### 2.9. Statistical Analysis

The results obtained during the multiple tests were archived and statistically analyzed using the following software: Microsoft Excel 2014 and Statistica 13.3 (StatSoft, Cracow, Poland). The Excel software was applied for calculating mean values with a standard deviation. For statistical analysis, response surface methodology (RSM) was used for fitting polynomial models as the quadratic equations of the tested characteristics depending on the variables used in the experiment.

## 3. Results and Discussion

### 3.1. Structure of TPS Moldings

**Figure 2:**
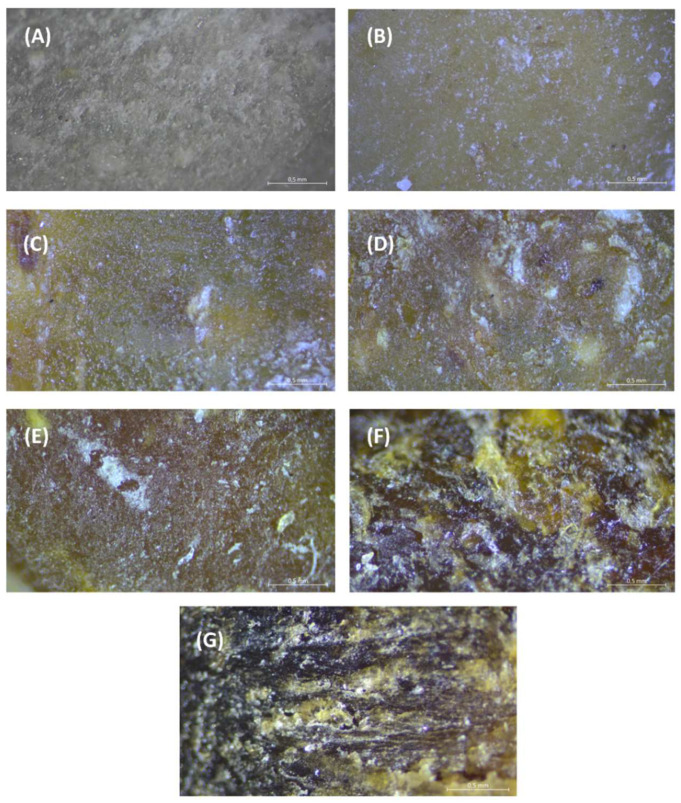
Structure of starch-based moldings processed at 140 °C with various durum wheat bran additions (magnification of ×2.5 with 8:1 zoom): (**A**) control sample; (**B**) 10% additive; (**C**) 20%; (**D**) 30%; (**E**) 40%; (**F**) 50%; (**G**) 60%.

Cross-sections of sample moldings processed at various temperatures and with diverse durum wheat bran additions were observed under the microscope. Our results indicate that the structure of samples injected at the processing temperature of 140 °C depend on the level of durum wheat bran addition (Figure 2).

The smooth, homogenous, and uniform internal structure shown in Figure 2A is formed by complete transformation and gelatinization of starch during the injection molding processing of TPS. Similar observations were found by Berti et al. [21] for tapioca starch films. Employing images observed under the optical microscope, as compared with our tested TPS samples they noted a homogeneous and continuous matrix. However, if they used rice bran particles (PRBs), the observations confirmed changes in the structure, increases in particle density, tensile strength, strain at break, as well as darkening of the matrix in agreement with color results when a higher amount of PRB was used, while the amount of bran was very low (0.3 g/100 g of slurry).

Application of durum wheat bran had a significant effect on structure changes in the sample moldings. If 10 or 20% of durum bran were used in the processed samples, only a few bran particles were visible on cross-sections of the tested moldings (Figure 2B,C, respectively). We noted that the structure was dense and compact similarly as in TPS maize starch samples. The glassy-like cross-section confirms the complete transformation and gelatinization of the starch in the processed samples. Application of 30 and 40% of durum bran in moldings recipes brought about the formation of a dense internal structure; however, in these samples (Figure 2D,E, respectively) we observed some empty spaces inside the moldings, which are seen as white parts on the mentioned pictures. This is the effect of the formation of empty air cells inside samples, probably caused by disruption of the compact starch-based structure by fibrous bran. However, biocomposites on the basis of thermoplastic starch filled by 25 and 40 wt% of wood or kenaf fibers also showed a two-phase character of the matrix and regular distribution of fibers in the matrix [22]. Moreover, Lu et al. [23] found no large agglomerates of the fillers and good adhesion between the matrix and fillers, which should play an important role in improving the mechanical performance. It should be noted that in their work, they used up to 40 wt% of ramie fibers nanofibrils which were well-diluted in the prepared biopolymer thin film.

Evident effect of structure disruption is visible in Figure 2F,G, which are structure pictures of moldings injected at 140 °C with the highest amount of durum wheat bran (50 and 60%, respectively). Such a high level of bran changed significantly the internal structure, which was more porous, rugged, and heterogeneous than that in the other observed samples with a lower bran addition level. Additionally, according to the effect of the bran addition on mechanical properties and elasticity of biopolymers, application of higher than 50% of durum bran in the composition of injected moldings made it less compact and lowered their resistance to breaking, but the samples were more elastic and less hard and brittle. One of the specificities of fibers as reinforcement materials is their poor dispersion characteristics in many thermoplastic melts, due to their hydrophilic nature [24]. We found that the bran particles were embedded into the gelatinized starch matrix, but there were empty spaces next to the bran fibers visible as yellow-white parts with many open structures of air bubbles being greatly shown in Figure 2G, where 60% of the bran level was applied in the TPS moldings.

Important changes were also observed when analyzing the effect of temperature on the structure of TPS moldings. Samples processed at higher temperatures (e.g., 160 and 180 °C) showed partial disruption of the internal structure of starch-based biopolymer due to a drying effect and increasing lightness of the surface of samples at such a high processing temperature. Better and more compact structure was observed when the injection molding temperature was not higher than 140 °C, as presented in Figure 2. However, for confirmation, deeper research is needed to evaluate the structure building during the processing of corn biopolymers reinforced by durum bran addition, the processing steps and conditions, because the major factors that modulate the properties of fiber–starch composites are related to fiber volume fraction, dispersion, size, shape, orientation and fiber-matrix adhesion [24].

### 3.2. Selected Mechanical Properties of TPS Moldings

The mechanical properties of products can be modified by using various fibrous additives [25,26,27,28]. Depending on whether the thermoplastic starch granulate is to be used in the production of rigid forms or foil, different types of starch should be used, including in terms of their botanical origin and the content ratio of amylose to amylopectin.

The conducted evaluation of selected mechanical properties of biopolymer moldings produced with the addition of durum wheat bran allowed us to determine the adequate composition of TPS granulate, as well as suitable manufacturing process parameters for the production of rigid forms using the technique of high-pressure injection molding. The results of this study may serve as a basis for the establishment of the optimum parameters in the production of thermoplastic starch and its blends, and consequently facilitate the development of comprehensive production technologies dedicated to manufacturing rigid biodegradable forms with the addition of waste raw materials such as durum wheat bran, and the application thereof in, e.g., the packaging industry.

Apart from altering the mechanical properties of the end product, fillers also serve a stabilizing function. One of the problems in TPS processing relates to the phenomenon of end product shrinkage, which can be largely eliminated by employing suitable fillers [29].

#### 3.2.1. Tensile Tests

**Figure 3:**
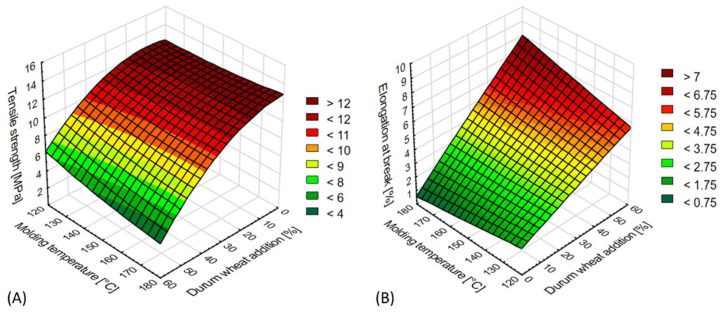
Selected mechanical properties of TPS moldings in the tensile test: (**A**) tensile strength (MPa), (**B**) elongation at break (%).

During the conducted tensile strength tests, it was observed that in each of the molding variants tested, the content of durum wheat bran and the bioplastic injection temperature significantly affected the mechanical properties of the products. Detailed results are presented in Figure 3A,B.

The tensile strength of the TPS moldings depended on the content of the durum wheat bran additive (Figure 3A). The recorded values were within the range from 3.43 to 14.63 MPa. For 10 and 20% additive content, no notable decrease was recorded relative to the control sample, indeed, in one configuration (20% additive, injection temperature 120 °C), the tensile strength was higher (14.42 MPa) than in the case of the sample with no additive (13.98 MPa). At the same time, in samples containing over 20% of the bran additive, a significant deterioration was observed in terms of this parameter. For example, the tensile strength of moldings containing 60% bran was four times lower relative to the control. The temperature of the high-pressure injection also significantly influenced the relevant property of samples containing over 30% bran, where the tensile strength decreased with increasing temperature of the process.

We observed that the elongation at break increased for higher durum wheat bran content and high-pressure injection temperature (Figure 3B). The obtained values were within the range from 0.80 to 8.53%. The highest value of elongation at break was noted for moldings containing 60% bran in the whole range of biomaterial injection temperatures.

Table 1 presents the fit functions for the respective response surface models in particular tests. It can be assumed that the above dependencies were significantly influenced by the form of the durum wheat bran itself, which is characterized by the presence of thick, long fibers at the granulation level of over 560 µm. The higher content of thick bran particles weakened the structure of the moldings, resulting in lower tensile strength, but also enabled better elongation ability, i.e., higher elasticity. The properties of starch–fiber nanocomposites are strongly determined by fiber concentration because at low fiber level a decrease in tensile strength is usually observed. This phenomenon can be connected with the dilution of the matrix and introduction of flaws at fiber ends, wherein a high stress concentration occurs causing breaking bonds between the fiber and matrix. At a high amount of fiber fraction, stress is more evenly distributed and a reinforcement effect is observed until a threshold is determined by inherent properties of cellulose fibers; to exceed this threshold, the cellulose fibers are flocculated and percolated, causing weak points in structures, and there is a critical volume to observe the effect of reinforcement on the matrix, which decreases with increasing strength of fibers [24]. Lu et al. [23] tested starch reinforced with ramie nanofibrils of glycerol-plasticized starch, and they found that composites with 5, 10, 25, and 40 wt% of ramie fibers increased tensile strength values from 2.8 to 6.9 MPa with a significant decrease in strain values from 92% to at least 10% of elongation at break when 40% of nanofibrils were used. However, in this work, the fibers were assessed via acid hydrolysis and this approach can change the fiber surface properties.

We found that during the extrusion cooking process of the thermoplastic starch, the bran fibers do not allow stable internal connections between the fibrous bran surface and starch-protein matrix. This effect can weaken the structure of the obtained materials. Research confirming this observation, i.e., loose and empty structures placed close to fiber parts in the inside, was presented by Wójtowicz [30]; hence, this can be the source of weakening of TPS materials enriched with durum bran addition.

As evidenced by results published in the literature, the type of filler used as functional additive can enhance or reduce specific properties of TPS products. Similar tensile strength correlations were observed for TPS elements by Diyana et al. [31], who analyzed manioc TPS with the addition of beeswax. However, in their tests, elongation at the break value was reduced. Girončs et al. [32] reported that increasing the content of sisal and hemp fibers improved (even two times compared with the control) the tensile strength of the product, while at the same time reduced elongation at break values. Wollerdorfer and Bader [33], in studying the addition of various raw materials, also reported a significant impact of the type and content of fibers on the mechanical properties and strength of high-pressure injection moldings fabricated from corn and wheat starch. In the experiments, various plant fibers including flax, jute, and oil palm fibers (up to 25%) were used, which increased (even four-fold) the tensile strength of the resulting reinforced materials. Ma et al. [34], in researching molded elements reinforced with cotton fibers, revealed correlations between the mechanical properties of TPS and the fiber content. When the fiber content was increased from 0 to 20%, the initial tensile strength was tripled and reached 15.16 MPa, while the elongation decreased from 105 to 19%.

#### 3.2.2. Bending Test

**Figure 4:**
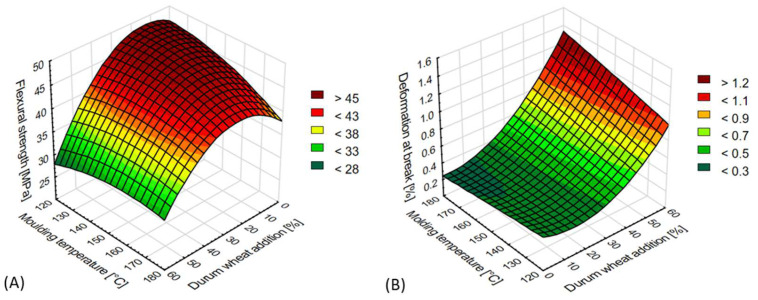
Selected mechanical properties of TPS moldings in the 3-point bending test: (**A**) flexural strength (MPa), (**B**) deformation at break (%).

The conducted bending tests indicated that the relevant mechanical properties were affected by the content of durum wheat bran and temperature of the injected biomaterials. The results are presented in Figure 4A,B.

The addition of up to 20% durum wheat bran yielded the maximum results in flexural strength tests for the tested TPS moldings. The obtained values ranged from 27.14 to 48.66 MPa (Figure 4A). When the added amount exceeded 20%, the flexural strength rapidly decreased, with the lowest results recorded at 60% bran content. The injection temperature during the moldings’ production also impacted this mechanical property, with the highest flexural strength observed in samples manufactured at the lowest injection temperatures. We saw that the higher the biomaterial injection temperature (for samples containing between 0 and 30% bran), the lower the flexural strength of the product. No significant impact of the injection temperature was observed in samples containing over 30% bran.

The amount of bran used in the blend and the temperature of starch-based materials injection also significantly affected the other bending property tested: deformation at break (Figure 4B). The values recorded in the tests ranged from 0.20 to 1.56%. The highest were obtained for moldings containing 60% durum wheat bran and with an injection-molded temperature of 180 °C. For most variants, lower injection temperatures correlated with lower deformation at break results. With decreasing content of durum wheat bran in the moldings, their deformation at break also decreased. This dependence was observed for a vast majority of the samples tested. A clearly increased extent of deformation was observed for moldings containing over 40% bran.

Table 2 presents the fit functions for the respective response surface models in particular tests. It can be assumed that, similarly to the tensile test, the parameters and physical characteristics of the durum wheat bran used in the study likely had a significant bearing on the tested properties, in particular, their degree of break-up (granulation) and amount of fibers. A higher content of thick and long bran fractions must have negatively influenced the structure of the produced moldings (hence, their lower flexural strength), while at the same time positively influencing the functionally important parameter of biomaterial deformation ability.

In the context of bending tests, the published experimental results indicate that in most cases, the use of a filler of plant origin (e.g., fibrous) in TPS resulted in an improvement of analyzed strength parameters. Our results are similar to those of Espinah et al. [35], who also demonstrated that the use of TPS composites reinforced with alpha-grass fibers (at between 5 and 35%) facilitated an increase in the flexural strength of injection-molded forms. Moreover, the positive impact of functional additives on the durability of various TPS products has been reported by other authors [31,36]. Indeed, the addition of cotton, cogon grass, and seaweed fibers in TPS products improves their flexural strength and deformation capacity.

### 3.3. Color of Starch-Based Moldings

**Figure 5:**
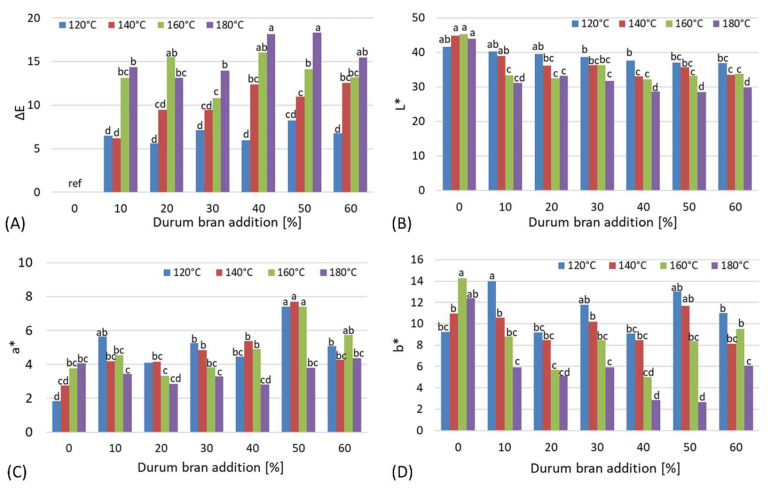
Color coordinates of durum wheat bran-enriched starch-based moldings: (**A**) ΔE—total color change index, (**B**) L*—lightness, (**C**) a*—red-green balance, (**D**) b*—yellow-blue balance; ^a–d^—different letters in each figure indicate significant differences between samples at *p* ≤ 0.5.

The color of a natural biopolymer may limit its practical usability due to consumer preferences, or suggest the type of more desirable and acceptable components or additives. The color coordinates of L*, a*, and b* and the total color change index of starch-based moldings enriched with durum wheat bran additives were evaluated for each sample surface. The results are presented in Figure 5A–D.

The total color change index clearly evidences visible differences between respective samples that were dependent both on the injection molding temperature and the level of durum wheat bran enrichment (Figure 5A). The lowest and insignificantly different values of ΔE were observed for samples processed at the lowest temperature of 120 °C compared with reference starch-based samples without additives. In almost all samples, the highest total color change index was observed for the highest injection temperature at all levels of bran enrichment. This suggests that intensive thermal treatment of biopolymer moldings at temperatures exceeding 120 °C may result in surface darkening, as confirmed by the recorded values of the L* coordinate, due to the Maillard reactions occurring at higher temperatures. Such changes in lightness were not observed in the control sample without bran addition (Figure 5B). Fiber-rich bran obtained from durum wheat is also a rich source of proteins that undergo Maillard reactions together with the simple sugars, thus leading to darkening of the material [20,37,38]. Additionally, bran is much darker than starch, so increasing the additive level naturally influenced lightness values, although such differences were mostly statistically insignificant, especially in the ranges from 10 to 30% and from 40 to 60% of bran added, showing homogenous groups within these two ranges.

At the same time, significant differences were observed relative to the injection temperature employed, especially between 120 and 180 °C, and between 160 and 180 °C, with durum wheat bran enrichment exceeding 30%. Measurements of the a* coordinate, describing changes from red if positive to green if negative, returned only positive values for durum wheat bran enriched moldings (Figure 5C). This means that all samples were characterized by a slightly red tint, which intensified with the increasing content of bran in the biopolymer composition. The highest values were observed for 50% of durum wheat bran added and injection temperatures between 120 and 160 °C. Higher temperature and higher bran content caused dryness of the surface and a less intensive red tint of the moldings. What is more, the b* values were positive in all the samples, indicating a slight yellow shade of the moldings because of the natural, yellow-brownish tint of the durum wheat bran used in the experiment. The most intensive yellowness was observed in moldings without the additives and in samples injected at the temperature of 120 °C (Figure 5D). Significant differences in terms of yellowness, showing a decrease in the light-yellow tint, were observed for increased injection temperatures; in all the bran-enriched samples, the lowest values of b* were recorded for samples processed at the highest temperature [39,40].

### 3.4. Analysis and Characteristics of Starch-Based Biopolymer Samples Using ART/FTIR Infrared Spectroscopy

In the next stage of the experiment, Attenuated Total Reflectance/Fourier Transform Infrared Spectroscopy (ART/FTIR) was employed to facilitate a more precise and detailed analysis, particularly at the molecular level, of the obtained corn starch-based biopolymer moldings.

**Figure 6:**
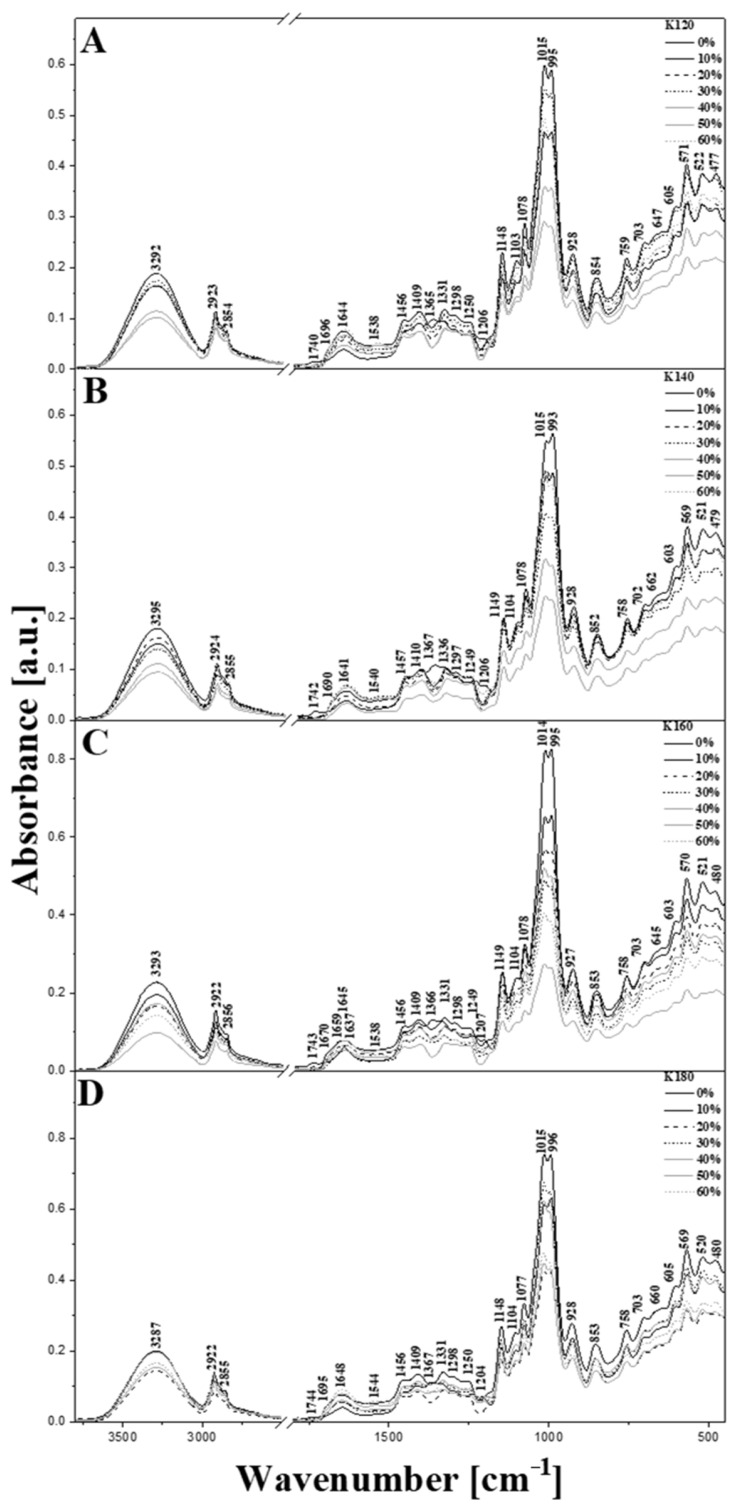
ATR/FTIR infrared spectra for the analyzed corn starch samples with variable percentage ratios of durum wheat bran addition in the range from 450 to 3750 cm^−1^. (**Panel A**) (K120): injection temperature of 120 °C; (**Panel B**) (K140): injection temperature of 140 °C; (**Panel C**) (K160): injection temperature of 160 °C; (**Panel D**) (K180): injection temperature of 180 °C.

In order to examine the nature of interactions between the starch and the additive used, FTIR spectroscopy was employed. The influence of the injection temperature on the product was also analyzed with the use of this technique. All spectra are presented in Figure 6. For better clarity, the vibrations corresponding to particular bands observed are provided in Table 3.

Based on our previous studies [41,42,43,44,45], the leftmost characteristic area of each spectrum (Figure 6) shows the maximum at ~3290 cm^−1^, assigned to the stretching vibrations of -OH (ν(-OH)) groups (both free and the hydrogen bound -OH groups cause bands in this region). Since the starch remains the main component of the biopolymers tested, this band most likely originates from -OH groups of amylose or amylopectin. The additional enhancement (broadening) of this band is caused by the presence of water and glycerol. Undoubtedly, the region of ~3290 cm^−1^ is occupied by signals resulting from various stretching vibrations of the hydroxyl groups. It is also worth mentioning that introduction of additives resulted in a notable decrease in intensity and further broadening of the band present in this region, which points at the increased number of intermolecularly hydrogen bonded -OH groups occurring as a result of interactions between the starch and the bran additive [46,47,48]. The decreased intensity of the band at 3290 cm^−1^ upon the addition of bran suggests that the affinity of search molecules to the additives used is higher compared with that of water. It is also in line with the fact that the presence of the additive affects results in an increase in the intensity of band at 1640 cm^−1^ assigned to the deformational vibrations of water molecules.

As seen in Figure 6, the intensity of vibrations in the region of 3290 cm^−1^ depends mainly on the content of durum wheat bran present in the blend, while the dependence on injection temperature is less pronounced. This may be related to the moisture content in the additive, or directly on the amount of additive. Based on the existing reports, this band may be notably shifted as a result of the growing tendency of the sample components to form intermolecular hydrogen bonds. An example of such a shift is visible in Figure 6D; however; in this particular case, it is more likely due to a high injection temperature and degradative processes which may take place in the sample.

The next highly informative area of the spectra corresponds to the stretching vibrations of C–H bonds and particularly those of the CH_2_ groups present in the structures of starch (mainly amylose) and the additive [47,48,49,50,51]. The maximum intensity of these vibrations is observed at ~2920 and 2850 cm^−1^. A notable deformation of bands in this range may indicate the substantial impact of injection temperatures on the molecular interactions in the studied starch-based biopolymers. As already mentioned, the deformation vibrations of hydroxyl groups are present as a moderately intensive band with the maximum at approx. 1640 cm^−1^ (Figure 6) [41,52,53]. It should also be noted that for this particular type of sample, the band in this region may originate from or may be enhanced by the stretching vibrations of C-C bonds. Moreover, these bands may correspond to the intermolecular hydrogen bonds between the starch and durum bran wheat additives.

The characteristic band at ~1740 cm^−1^ may be assigned to the vibrations of the carbonyl groups present in protein components [54]. It is, however, interesting that the putative carbonyl band is most intensive in the control sample not containing any bran, and is probably related to the additive. Each sample tested shows a relatively weak intensity band, characteristic for degradation processes which likely take place in the injection molded sample. The relatively low intensity of this band evidences the good quality of the prepared samples. However, increased content of durum wheat bran in the blend was found to induce a deterioration of the bands’ intensity, supporting the hypothesis of the formation of hydrogen bonds between the main units of the starch biopolymer structures. Nonetheless, the band provided a fairly good marker for changes taking place in the biopolymer systems analyzed.

The analysis of the fingerprint region provides more valuable information, especially in that this region consists of a large number of prominent bands such as the deformative vibrations of C-H (~1370 cm^−1^) [52,55], CH_2_ (~1410 cm^−1^) [43], and C–OH bonds. All these groups are present in the structure of starch, mainly in amylopectin, though this area is also characteristic of protein additives coming from durum wheat bran. Other notable and highly informative bands are observed at ~1540 and 1460 cm^−1^ and correspond to the vibrations of C-C bonds [41,44]. These vibrations mainly come from the starch-building components, but can be reinforced by the addition of proteins. Interestingly, bands within the range of 1440–1240 or below 940 cm^−1^ tended to gain intensity with the increased amount ratio of durum wheat bran and were irrelevant to the injection temperature. This evidenced an increase in the content of ingredients such as proteins or gluten from wheat bran in the analyzed moldings. The extent of the mentioned increase was also reflected by the intensity changes in bands at approx. 1015 and 995 cm^−1^ (discussed below).

The next important area contains a signal with the maximum at ~1150 cm^−1^, which originates mainly from the ring stretching vibrations in the main component of the samples. This was enhanced by the stretching vibrations of C-O bonds, as well as by the C-O-C (ν(C-O-C)) moieties of amylose and amylopectin molecules [45,49]. Another set of vibrations is represented by the bands with the maxima at ~1015 and 995 cm^−1^, which correspond primarily to the C-O stretches that also are very characteristic for these types of biopolymer structure [41,56]. These bands are enhanced (overlapped) with the stretching vibrations of the C-O-H system.

The observed changes in the intensity ratio of 1015 and 995 cm^−1^ bands are relevant to C-O and C-O-C stretching vibrations in the anhydroglucose rings present in the starch molecules. These changes point toward the weakening of hydrogen bonding between the starch molecules resulting from the presence of the bran additive, as confirmed by mechanical properties testing. Furthermore, an increased amount of additive results in an overall intensity decrease in both bands mentioned, which additionally supports the hypothesis of the hydrogen bonds being weakened between the starch molecules. It should be highlighted that despite the modifications of adding varying amounts of durum wheat bran or varying injection molding temperature, the shapes of bands did not significantly differ from the reference spectra (black line in each Figure 6 panel). In contrast, a clearly visible change occurred in the area ratio of the 995 and 1015 cm^−1^ bands (Figure 6) [43,57]. This ratio change corresponds to the increase in additive content in the primary biopolymer structure, and to enhanced interactions between the C-O groups and the hydrogen bonds formed. Herein, the area’s intensity was primarily dependent on the stretching vibrations in the C-O-C system present in the starch structure [41,57].

It is also worth mentioning, that similar intensity changes are observed for the bands present between 940 and approx. 470 cm^−1^. These bands are associated mainly with the vibrations of the sugar (carbohydrates) moieties constituting the structure of the biopolymer’s main component [41,44,54,58]. The intensity changes observed in this region are quite significant and in most cases show a maximum at approx. 571 cm^−1^, i.e., in the range where slight enhancement by the deformation vibrations of hydroxyl groups may be present. The vibrations characteristic of these groups corresponds in this area primarily to the formation of hydrogen bonds between biopolymer units. Moreover, they are related to the vibrations of α-1,4-glycosidic and α-1,6-glycosidic bonds between monomers present in the structure of starch. These vibrations are characteristic for bonds constituting the main structural units in corn starch, such as amylose and amylopectin.

The injection temperature was another parameter that affected the IR bands of the biopolymers obtained and several bands demonstrated correlation with injection temperature. In this context, a noticeable decrease in the intensities of bands at ~3290 or 1140–940 cm^−1^ was observed.

It is worth emphasizing that the changes in IR bands that are related to the injection temperature point indicate that the bran fibers do not allow stable bonding between the surface of the bran fiber and the starch-protein matrix. This in turn may affect in structural weakening of the material obtained and manifests in the vibrations characteristic of the hydrogen bonds. These observations are confirmed in other studies [46,47,48].

The IR spectroscopic data clearly points to the formation of hydrogen bonding between the starch and the additive, which are notably stronger compared with those of intra- and intermolecular hydrogen bonds occurring in the starch in the absence of additives. This results in a plasticization of the material obtained and is evidenced by the changes in intensity of bands below 900 cm^−1^, which are characteristic of the α-1,6-glycosidic and α-1,4-glycosidic bonds mentioned.

## 4. Conclusions

During the study, it was observed that the amount the durum wheat bran additive used, as well as the temperature of high-pressure injection molding both had a significant impact on the structure and mechanical properties of the TPS moldings subjected to mechanical strength testing. In summary, the moldings produced with 10 and 20% durum wheat bran enrichment and at higher injection temperatures were characterized by higher tensile strength and greater elongation at break, compared with the control samples. This notion was confirmed by structure observations. In the bending test, the addition of durum wheat bran up to 20% had influence on TPS moldings that resulted in the highest flexural strength—even higher than in the case of control samples. The maximum strength values were recorded for samples obtained at the lowest temperatures. The highest deformation at break results were recorded for moldings with the maximum bran content, molded at 180 °C.

It was further observed that the color of the corn starch-based biopolymer products also depended on the ratio of durum wheat bran enrichments, as confirmed by structure analysis, as well as the injection temperature. At the highest ratio of bran addition and for high injection temperature, sample surfaces were observed to be dried out, and the intensity of color changes was reduced, but the high values of the color total change index nonetheless evidence the impact of the analyzed variables.

The application of FTIR spectroscopy allowed for a clear identification of several marker bands denoting the impact of durum wheat bran addition and injection temperature on the production of biopolymer moldings. In this context, monitoring of changes in bands at 3292 and 1644 cm^−1^, and those within the region of 1460–1240 cm^−1^ and below 900 cm^−1^ is particularly useful. Moreover, the changes in intensity ratio of bands at 1015 and 995 cm^−1^ are equally important. They correspond well with the amount of additive used and with the injection temperature applied and hence may be considered as markers of interactions affecting plasticization of the material obtained. More specifically, they reflect the weakening of intermolecular hydrogen bonding in starch upon an increase in the amount of additive. The changes in respective bands can be used to easily identify the additive used in respective samples. At the same time, even at the relatively high temperatures used in the production of the tested biopolymer, no clear degradational changes could be observed at the molecular level, which confirmed the high quality of the products.

The obtained results in terms of selected strength, color, and structural properties of the starch-based moldings are significant in the context of the potential usability of durum wheat processing waste in the production of TPS and other environmentally friendly materials. A more detailed spectroscopic analysis of samples aiming at the identification of even more characteristic marker bands will be the subject of our future studies.

## Figures and Tables

**Table 1:** Response surface fit models for selected mechanical properties of TPS moldings in the tensile strength test, relative to the content of durum wheat bran and high-pressure injection temperature.

Property	Fit Function for the Response Surface Model
Tensile strength (MPa)	17.9994 + 0.14x − 0.0864y − 0.0024x^2^ − 0.0008xy + 0.0003y^2^
Elongation at break (%)	4.967 − 0.057x − 0.0283y − 6.9685 × 10^−5^x^2^ + 0.001xy + 2.1464 × 10^−5^y^2^

x—durum wheat bran addition (%), y—injection molding temperature (°C).

**Table 2:** Response surface fit models for selected mechanical properties of TPS moldings in the flexural strength test, relative to the content of durum wheat bran and high-pressure injection temperature.

Property	Fit Function for the Response Surface Model
Flexural strength (MPa)	20.0532 − 0.1552x + 0.474y − 0.0093x^2^ + 0.0033xy − 0.0021y^2^
Deformation at break (%)	0.5338 − 0.026x − 0.0003y + 0.0003x^2^ + 0.0001xy − 7.1429 × 10^−6^y^2^

x—durum wheat bran addition (%), y—injection molding temperature (°C).

**Table 3:** The positioning of the maxima of absorption bands ATR/FTIR and corresponding vibrations for selected sampling in the spectral range of 3750–450 cm^−1^. The spectra were measured for the additive ratio of 60%.

Maximum Position (cm^−1^)				Types and Origin of Vibrations
K120	K140	K160	K180	
3292	3295	3293	3287	ν_st._(-OH) in the structure of cellulose and with H_2_O
2923	2924	2922	2922	ν_s+as_ (C-H) in CH_2_
2854	2855	2856	2855	
1740	1742	1743	1744	ν_m_ (C=O)
1644	1641	1645	1648	ν (C=O) and δ_m_ (O-H) in cellulose or adsorbed H_2_O
1538	1540	1538	1544	ν_m_ (C-C) and δ(CH_2_)
1456	1457	1456	1456	
1409	1410	1409	1409	δ(CH_2_)
1365	1367	1366	1367	ν (C-H) + δ(C-OH) + ν (C-C)
1331	1336	1331	1331	
1298	1297	1298	1298	
1250	1249	1249	1250	ν (CH_2_)
1206	1206	1207	1204	
1148	1149	1149	1148	ν (C-O-C) entire ring in the structure of starch + ν (C-C)
1103	1104	1104	1104	ν (C-O-H)
1078	1078	1078	1077	
1015	1015	1014	1015	ν_st._ (C-O-H) and ν_st._ (C-O)
995	993	995	996	
928	928	927	928	ν (C-O) and ν_w_ (C-C) in the starch ring and CH_2_ in the ring and C-OH out of plane bending *β-*linkage of cellulose ν (C-O-C) + δ (C-O-C)
854	852	853	853	
759	758	758	758	
703	702	703	703	
647	662	645	660	ring breathing
605	603	603	605	
571	569	570	569	
522	521	521	520	
477	479	480	480	

ν—stretching, δ—deformation, s—symmetrical, as—asymmetric, st.—strong, w—weak, m—medium.

## Data Availability

Samples of each material used in this study, namely, the TPS moldings with durum wheat addition are available on request from the Department of Thermal Technology and Food Process Engineering laboratory. The datasets generated during and/or analyzed during the current study are available from the corresponding author on reasonable request.
